# DualCDM: Dual-Domain Conditional Diffusion for SAR-to-Optical Translation with Spatial–Frequency Correlation and Adaptive Feature Recalibration

**DOI:** 10.3390/s26134183

**Published:** 2026-07-02

**Authors:** Yaobin Ma, Hossein Aghababaei, Ling Chang, Jingbo Wei

**Affiliations:** 1 Institute of Space Science and Technology, Nanchang University, Nanchang 330031, China; 2 Faculty of Geo-Information Science and Earth Observation, University of Twente, 7500 AE Enschede, The Netherlands

**Keywords:** SAR-to-optical, image translation, dual-domain, conditional diffusion model

## Abstract

Translating Synthetic aperture radar (SAR) images into optical images is intrinsically ill-posed because microwave backscatter and optical reflectance describe different physical properties of the observed scene. Although frequency-domain modeling has been introduced into diffusion-based translation, existing methods mainly rely on independent weighting of individual Fourier coefficients and provide limited modeling of interactions among neighboring frequencies and feature channels. To address this limitation, we propose dualCDM, a conditional diffusion model that jointly exploits spatial- and frequency-domain representations. In the diffusion backbone, a spatial-frequency hybrid residual block (SFHRB) combines a spatial convolution branch with complex-valued convolution in the Fourier domain. The complex convolution aggregates neighboring Fourier coefficients across all input feature channels, enabling local cross-frequency and cross-channel modeling, while its response is modulated by the diffusion timestep. In the SAR conditional encoder, an adaptive frequency-domain feature recalibration block (AFFRB) predicts input-dependent real-valued gains from magnitude and trigonometric phase representations of intermediate GRD features. These gains adaptively recalibrate the complex frequency responses without introducing an additional phase shift, while the residual connection preserves the original conditional information. A dual-domain objective further constrains both the predicted diffusion noise and the one-step optical reconstruction in the spatial and frequency domains. We also construct the S1S2 dataset using 16-bit Sentinel-2 reflectance data, retaining the original 0–10,000 value range and including the near-infrared band. Experiments on SEN1-2 and S1S2 show that dualCDM improves radiometric accuracy, spectral consistency, and structural preservation over six representative methods. Paired statistical tests further confirm significant improvements over the strongest competing method across all six evaluation metrics on both datasets.

## 1. Introduction

Optical and synthetic aperture radar (SAR) sensors provide complementary observations of the Earth surface. Optical imagery records spectral reflectance and spatial appearance in an intuitive form, but its availability is often limited by clouds and atmospheric effects [1]. SAR provides all-weather and day-and-night observations, but its side-looking imaging geometry, coherent scattering mechanism, and speckle make visual interpretation difficult. SAR-to-optical image translation, therefore, offers a way to generate optical-like representations from SAR observations, supporting visual interpretation and providing auxiliary information when cloud-free optical acquisitions are unavailable [2]. However, this translation remains challenging because SAR backscatter and optical reflectance are governed by substantially different imaging mechanisms.

Early SAR-to-optical translation methods mainly relied on convolutional neural networks to extract and recombine spatial features. Fu and Zhang [3] formulated remote sensing image translation as a sequence of image understanding, object transformation, and representation. Generative adversarial networks (GANs) subsequently became widely used because adversarial learning can improve the visual realism of generated images [4,5,6,7,8]. More recent studies have introduced Transformer architectures [9,10] and diffusion models [2,11,12] to improve long-range modeling and generation stability. A comprehensive review of this rapidly developing field was provided by Xiong et al. [13].

Frequency-domain modeling has recently been introduced to improve the representation of textures, boundaries, and other fine-scale structures. For example, Qin et al. [14] proposed a spatial-frequency diffusion framework that jointly processes target features in the two domains. This direction is also motivated by the spectral bias of neural networks, which tend to learn low-frequency components earlier than high-frequency components [15].

Modeling the correlations among spectral intensities and channels in the frequency domain helps capture a more comprehensive data distribution, properties that has yet to be exploited by diffusion translation networks. Existing frequency-domain modules apply pointwise or channel-wise weighting to individual Fourier coefficients. Although such operations can recalibrate spectral responses, they provide limited interaction among neighboring frequency components and feature channels. This limitation is important because spatial structures are generally represented by groups of correlated Fourier coefficients rather than isolated spectral points. Edges, textures, and repeated patterns produce locally dependent responses within spectral neighborhoods [16]. Moreover, applying the Fourier transform independently to each feature channel does not eliminate inter-channel dependence, which remains encoded in the complex-valued spectral coefficients. Effective frequency-domain modeling should, therefore, capture both local cross-frequency interactions and cross-channel correlations while preserving the complex relationships among Fourier coefficients.

The quality of the SAR conditional features presents another challenge. Speckle-related fluctuations can interfere with the extraction of boundaries, regional patterns, and strong scattering structures that provide important guidance for optical image generation. A common strategy is to apply an external despeckling filter to the SAR input, such as probabilistic patch-based filtering [14]. However, this preprocessing is performed independently of the translation objective and follows a predetermined filtering rule, which may remove structurally useful details together with noise-related variations. These limitations leave open how to reduce noise-related interference without sacrificing the structural information required for SAR-to-optical translation.

To address these limitations, we propose dualCDM, a conditional diffusion framework that combines spatial-frequency feature modeling, adaptive frequency-domain recalibration of SAR conditional features, and dual-domain supervision. In the diffusion backbone, SFHRB uses complex-valued convolution to model local cross-frequency and cross-channel correlations, while a parallel spatial branch preserves local image context. In the SAR conditional encoder, AFFRB learns input-dependent gains to recalibrate intermediate frequency-domain feature responses. A spatial-frequency objective further constrains both noise prediction and one-step optical reconstruction. We also construct a native-scale multispectral dataset to evaluate SAR-to-optical translation beyond conventional 8-bit RGB settings.

The main contributions are summarized as follows:

(1) Local cross-frequency and cross-channel modeling: We introduce SFHRB, which combines a spatial convolution branch with a complex-valued convolution in the Fourier domain. Unlike independent per-frequency reweighting, the complex-valued convolution jointly processes the real and imaginary components of neighboring Fourier coefficients while mixing information across all input channels.
(2) Adaptive frequency-domain recalibration of SAR conditional features: We introduce AFFRB into the SAR conditional encoder to adaptively modulate the Fourier-domain responses of intermediate real-valued GRD features. The module predicts input-dependent real-valued gains from the spectral magnitude and trigonometric phase representations and applies them to the complex Fourier coefficients.
(3) Dual-domain supervision: We constrain the predicted diffusion noise and the one-step optical estimate in the frequency domain in addition to the standard spatial-domain noise loss.
(4) Native-scale multispectral benchmark: We construct the S1S2 dataset using Sentinel-2 reflectance values retained on the 0–10,000 scale and including the near-infrared (NIR) band.

## 2. Related Work

In the context of remote sensing image translation, some studies have demonstrated the effectiveness of GANs in translating SAR images into optical images. Enomoto et al. [4] implemented SAR-to-optical image translation using conditional GAN. Reyes et al. [5] conducted image translation experiments using cycle-consistent GAN on both high-resolution data (TerraSAR-X and ALOS PRISM) and low-resolution data (Sentinel-1 and Sentinel-2). To improve the image quality and training stability, several new GAN-based methods were proposed. Li et al. [6] developed a modified cGAN with strong constraints based on structural similarity and L1 norm. Zhang et al. [7] introduced a feature-guided method that effectively utilizes the diversity of structural and texture features. A reciprocal adversarial network scheme that utilizes cascaded residual connections and a hybrid L1-GAN loss function was proposed by Fu et al. [8].

In addition to GANs, Transformer has also been used for the translation. Kong et al. [9] proposed an encoder-decoder generator based on the SWIN Transformer. Wang et al. [10] proposed a hybrid conditional GAN that combines the advantages of convolutional neural network (CNN) and Vision Transformer, enhancing image fidelity by incorporating a classification loss.

Central to these studies, diffusion models [17] have recently demonstrated excellent capabilities in generation tasks. By learning specific data distributions, diffusion models generate high-quality images and outperform GANs [18]. For the SAR-to-optical translation task, diffusion models have been used in [2,11,12,14]. Furthermore, ref. [19] proposed to use the Brownian Bridge diffusion model by modelling image translation tasks as a stochastic Brownian Bridge process.

## 3. Methodology

### 3.1. Network Structures

**Figure 1:**
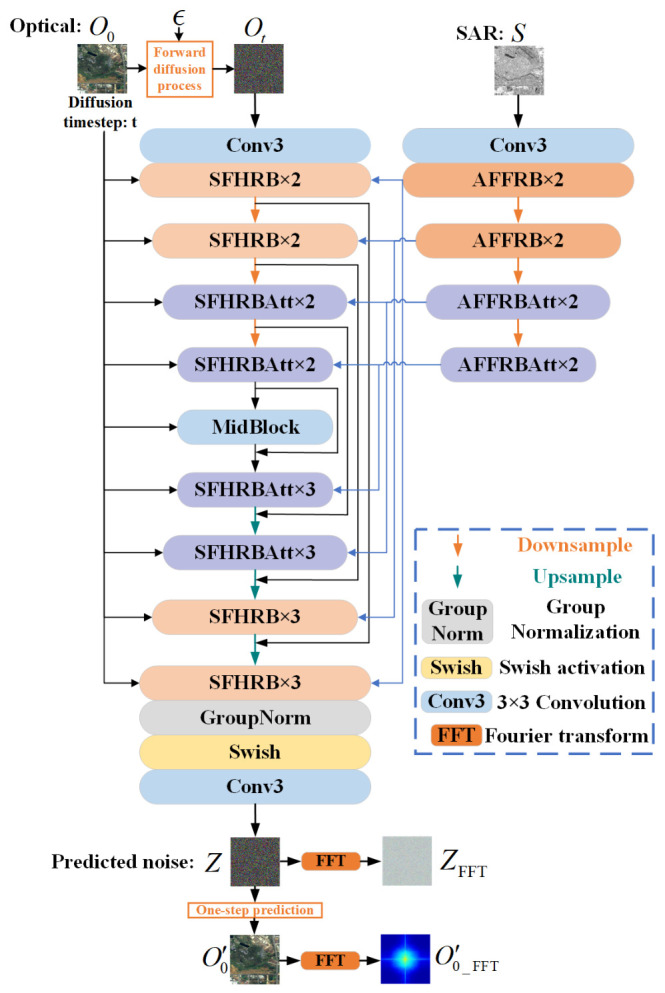
Architecture of the dual-domain conditional diffusion models (dualCDM).

The architecture of the proposed dual-domain conditional diffusion model is illustrated in Figure 1. It consists of a diffusion backbone and a SAR conditional encoder. The diffusion backbone follows a U-Net encoder–decoder architecture and predicts the noise added to the optical image at diffusion timestep *t*. In parallel, the conditional encoder extracts multi-scale structural and textural features from the SAR observation. These conditional features are injected into the corresponding resolution levels of the diffusion backbone through lateral connections, thereby providing scene-dependent guidance for noise prediction. Long skip connections between the encoder and decoder of the diffusion backbone preserve spatial information and facilitate the fusion of shallow structural details with deeper semantic features.

In the diffusion backbone, the noisy optical image $O_{t}$ is first projected into a 128-channel feature space using a 3×3 convolution (Conv3). The backbone contains four resolution levels indexed by l∈0,1,2,3, with spatial resolutions of $H/2^{l}\times W/2^{l}$ and channel dimensions of 128, 256, 384, and 512, respectively. Each encoder level contains two consecutively stacked residual blocks, denoted by ×2 in Figure 1, followed by downsampling except at the deepest level. The decoder follows the reverse multi-scale hierarchy and progressively upsamples the features while fusing them with the corresponding encoder features through skip connections. Each decoder level contains three consecutively stacked residual blocks, denoted by ×3, because an additional block is used to refine the fused decoder and skip-connected features. The two shallow resolution levels use SFHRB, whereas the two deeper and lower-resolution levels use SFHRBAtt. SFHRBAtt is the attention-enhanced variant of SFHRB and consists of an SFHRB followed by an AttnBlock. Self-attention is, therefore, applied only at the lower-resolution levels, where long-range dependencies can be modeled with lower memory consumption and computational cost. Finally, GroupNorm, Swish activation, and a 3×3 convolution are applied to generate the predicted noise *Z*, which has the same spatial resolution and number of channels as $O_{t}$.

The SAR conditional encoder adopts the same four-level resolution hierarchy and channel configuration as the diffusion encoder. The SAR image *S* is first projected into the feature space through a 3×3 convolution. At the two shallow resolution levels, the conditional features are extracted using AFFRB, whereas the two deeper levels employ AFFRBAtt. AFFRBAtt is the attention-enhanced variant of AFFRB and is composed of an AFFRB followed by an AttnBlock. Similar to the diffusion encoder, each resolution level contains two consecutively stacked blocks, denoted by ×2. The resulting conditional features are injected into the corresponding encoder and decoder stages of the diffusion backbone through lateral connections. Throughout Figure 1, the notation ×N indicates that *N* blocks of the specified type are consecutively stacked at the same spatial resolution and channel dimension.

#### 3.1.1. SFHRB Module

**Figure 2:**
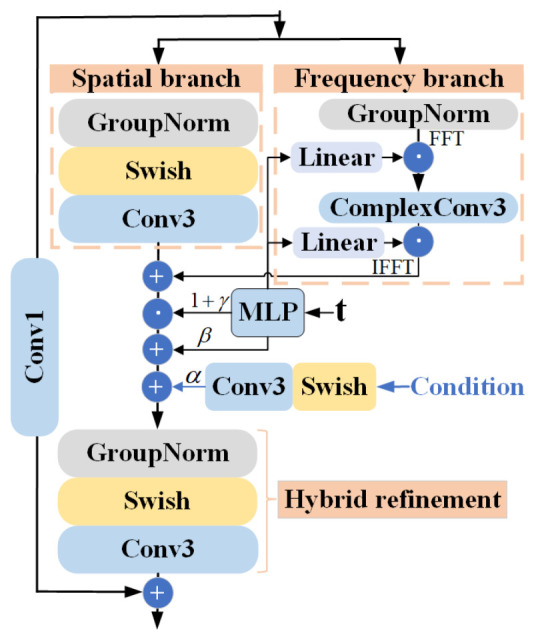
Structure of the spatial-frequency hybrid residual block (SFHRB).

To preserve local spatial textures while improving frequency-domain representation, we design SFHRB based on the standard diffusion residual block. As shown in Figure 2, it contains a spatial convolution branch operating in the spatial domain and a parallel complex-valued convolution branch operating in the Fourier domain. The frequency branch is further modulated by the diffusion timestep embedding. Unlike pointwise spectral weighting, which processes frequency locations independently [14,20], the complex-valued convolution aggregates neighboring frequencies across multiple channels, enabling local cross-frequency and cross-channel modeling.

Given the input feature map $x\in R^{B\times C_{\mathrm{in}}\times H\times W}$, where *B* denotes the batch size, $C_{in}$ is the number of input channels, and H×W represents the spatial resolution, we further consider the timestep embedding vector $t\in R^{B\times C_{t}}$ and the SAR conditional feature $c\in R^{B\times C_{\mathrm{cond}}\times H\times W}$. The spatial-domain branch applies normalization, nonlinear activation, and a 3 × 3 convolution to capture local spatial context,

(1) $$h_{s}=\mathrm{Conv}3\left(\phi \left(\mathrm{GN}(x)\right)\right)\in R^{B\times C_{\mathrm{out}}\times H\times W},$$

where $\mathrm{GN}\left(\cdot \right)$ denotes group normalization (GroupNorm), $\phi \left(\cdot \right)$ denotes the Swish activation function (Swish), and $\mathrm{Conv}3\left(\cdot \right)$ denotes a 3 × 3 convolution.

In parallel, the frequency-domain branch first applies a fast Fourier transform (FFT) to the normalized input features to obtain a complex spectral representation,

(2) $$H_{C_{\mathrm{in}}}\left(\omega \right)=F\left(\mathrm{GN}(x)\right)\in C^{B\times C_{\mathrm{in}}\times H\times W},$$

where $F\left(\cdot \right)$ denotes FFT, and $\omega =\left(\omega_{x},\omega_{y}\right)$ denotes the frequency-domain coordinates.

During the forward diffusion process, the intensity of injected random noise varies across timesteps, causing the statistical properties of features in the frequency domain to change accordingly. To adapt to the timestep-dependent spectral distribution and enable adaptive spectral recalibration, the timestep embedding vector *t* is linearly projected into a complex-valued modulation vector, which is used to modulate the input and output channels of the spectral features,

(3) $$g_{in}\in C^{B\times C_{\mathrm{in}}},g_{out}\in C^{B\times C_{\mathrm{out}}},$$

where $C_{\mathrm{out}}$ is the number of output channels.

First, complex-valued channel modulation is applied to the input spectral features, i.e., each channel of each sample is multiplied by a complex-valued scaling factor shared across spatial locations, thereby recalibrating the spectral response of each channel,

(4) $${\tilde{H}}_{C_{\mathrm{in}}}\left(\omega \right)=H_{C_{\mathrm{in}}}\left(\omega \right)⊙g_{\mathrm{in}},$$

where ⊙ denotes element-wise product.

Then, a complex-valued convolution is performed in the frequency domain,

(5) $$Y_{C_{\mathrm{out}}}\left(\omega \right)=\sum_{C_{\mathrm{in}}}\left({\tilde{H}}_{C_{\mathrm{in}}}{*}_{\omega }W_{C_{\mathrm{out}},C_{\mathrm{in}}}\right)\left(\omega \right),$$

where ${*}_{\omega }$ denotes the two-dimensional discrete convolution with respect to the frequency-domain coordinates ω, and $W_{C_{\mathrm{out}},C_{\mathrm{in}}}$ is the complex-valued convolution kernel that connects the input channel $C_{\mathrm{in}}$ to the output channel $C_{\mathrm{out}}$ (with a kernel size of 3 × 3 in this work).

Subsequently, complex-valued channel modulation is applied again to the output spectral features,

(6) $${\tilde{Y}}_{C_{\mathrm{out}}}\left(\omega \right)=Y_{C_{\mathrm{out}}}\left(\omega \right)⊙g_{\mathrm{out}}.$$

Finally, an inverse FFT (IFFT) is applied to return to the spatial domain, and the real part is taken as the output of the frequency-domain branch,

(7) $$h_{f}=ℜ\left(F^{-1}\left(\tilde{Y}\right)\right)\in R^{B\times C_{\mathrm{out}}\times H\times W},$$

where $F^{-1}\left(\cdot \right)$ denotes IFFT, and $ℜ\left(\cdot \right)$ denotes the real part.

Specifically, the timestep embedding *t* is processed through a multilayer perceptron (MLP) to predict a per-channel scaling factor $\gamma \in R^{B\times C_{\mathrm{out}}}$ and bias $\beta \in R^{B\times C_{\mathrm{out}}}$:

(8) $$\begin{array}{cc} \left[\gamma ,\beta \right] & =\mathrm{Split}(\mathrm{MLP}(t)),\\ h_{1} & =\left(h_{s}+h_{f}\right)⊙\left(1+\gamma \right)+\beta . \end{array}$$

In addition, the SAR image is used as a conditional feature and mapped to $C_{\mathrm{out}}$ via a 3 × 3 convolution, and then injected into the main branch with a learnable coefficient α,

(9) $$h_{2}=h_{1}+\alpha \cdot \mathrm{Conv}3\left(\phi \left(c\right)\right).$$

The fused feature $h_{2}$ is further refined through hybrid refinement, and then added to the original input feature *x* via a skip connection to produce the final output,

(10) $$y=x+\mathrm{Conv}3\left(\phi \left(\mathrm{GN}(h_{2})\right)\right).$$

#### 3.1.2. AFFRB Module

**Figure 3:**
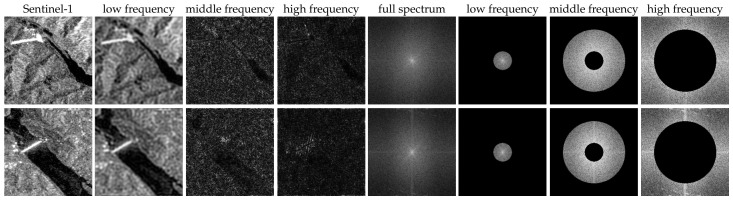
Visual comparison of original Sentinel-1 GRD images, their frequency components (low, middle, high), and corresponding magnitude spectrums.

As shown in Figure 3, the Sentinel-1 GRD intensity image is decomposed into low-, middle-, and high-frequency components for visualization. Specifically, the centered Fourier spectrum is divided using two radial thresholds, $R_{1}$ and $R_{2}$, and the corresponding components are reconstructed through inverse Fourier transform. The low-frequency component mainly retains the large-scale spatial layout, overall object outlines, and slowly varying background. In contrast, granular fluctuations and fine-scale textures become increasingly evident in the middle- and high-frequency components, indicating that these frequency ranges are more sensitive to speckle-related variations. Nevertheless, structurally relevant information, including sharp boundaries, strong gradients, and point-like scatterers, is also present in these frequency ranges. Therefore, directly suppressing the middle- or high-frequency components may remove both noise-related fluctuations and useful structural details. The fixed frequency decomposition shown in Figure 3 is used only for visualization and is not explicitly employed in the proposed network.

**Figure 4:**
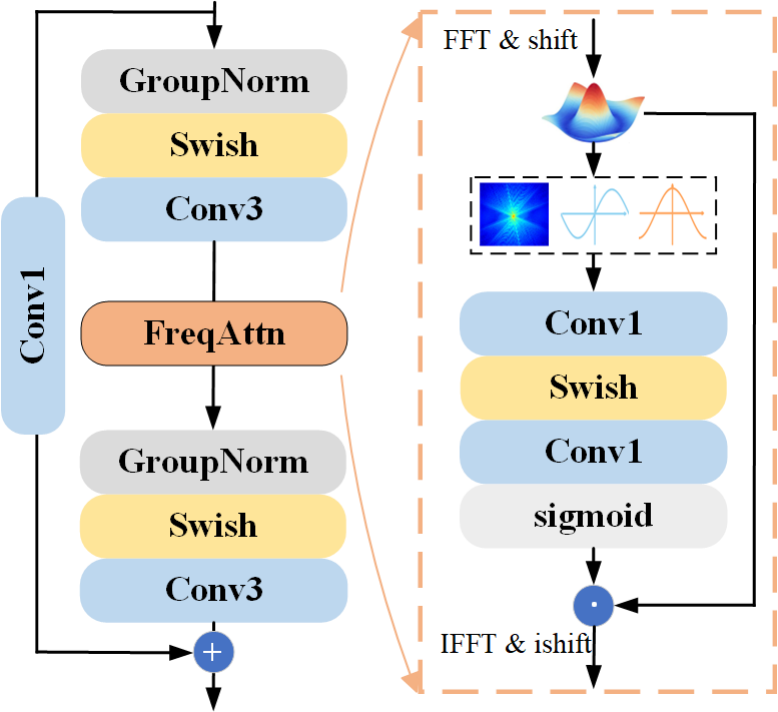
Structure of the adaptive frequency-domain feature recalibration block (AFFRB).

This observation motivates an adaptive frequency-domain feature recalibration strategy that selectively attenuates noise-dominated responses while preserving structurally informative frequency components. To implement this strategy, we propose a frequency-domain adaptive attention module, termed FreqAttn, and embed it into the residual blocks of the SAR conditional encoder to form AFFRB. Rather than dividing the feature spectrum into predefined frequency bands, FreqAttn predicts a continuous, input-dependent gain for each feature channel and frequency location. The detailed architecture of AFFRB is illustrated in Figure 4.

Given the intermediate feature $h_{sar}\in R^{B\times C\times H\times W}$ obtained after the first convolution, FreqAttn first applies an FFT to it and performs spectrum centering,

(11) $$H_{s}=S\left(F\left(h_{sar}\right)\right)\in C^{B\times C\times H\times W},$$

where S(·) denotes the spectrum centering operation.

Then, the magnitude and phase are extracted from $H_{s}$ to construct a joint feature,

(12) $$Z=\mathrm{concat}\left[M,\cos\left(\Phi \right),\sin\left(\Phi \right)\right]\in R^{B\times 3C\times H\times W},$$

where $M=\left|H_{s}\right|$ denotes the magnitude and $\Phi =∠H_{s}$ denotes the phase. To avoid representation ambiguity and numerical instability caused by the phase discontinuity at $\left[-\pi ,\pi \right]$, we apply a trigonometric encoding to the phase and use cos(Φ) and sin(Φ) as its representation.

The resulting joint feature *Z* is then fed into a bottleneck network composed of two consecutive 1 × 1 convolutions, which maps it to frequency-domain gain with the same number of channels as the original spectrum features $H_{s}$,

(13) $$L\;=\;\sigma \;\left(\;\mathrm{Conv}1^{\left(2\right)}\left(\phi \left(\mathrm{Conv}1^{\left(1\right)}\left(Z\right)\right)\right)\;\right)\;\;\in \;R^{B\times C\times H\times W},$$

where $\sigma \left(\cdot \right)$ denotes the sigmoid function. To avoid amplifying noise and reduce the risk of numerical instability, we constrain the frequency-domain gain *L* to the range $\left(0,1\right)$, so that it only attenuates rather than amplifies.

The gain *L* is used to multiplicatively modulate the spectral features on a per-channel and per-frequency basis, adjusting only the magnitude distribution while keeping the phase unchanged,

(14) $$H_{s\_att}=H_{s}⊙L.$$

Finally, the filtered spectrum is mapped back to the spatial domain via inverse centering and an IFFT, and its real part is taken as the output of FreqAttn,

(15) $$h_{out}=ℜ\left(F^{-1}\left(S^{-1}\left(H_{s\_att}\right)\right)\right)\in R^{B\times C\times H\times W}.$$

#### 3.1.3. Auxiliary Modules

**Figure 5:**
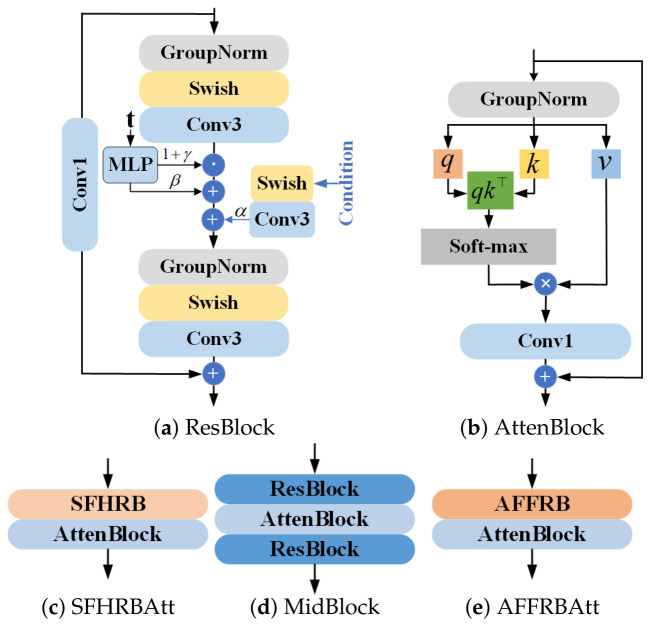
Other fundamental modules in the proposed diffusion model.

The other basic modules in the diffusion model are illustrated in Figure 5. The residual block (ResBlock in Figure 5a) consists of two convolutional layers, each followed by group normalization (GroupNorm) and a Swish activation. The diffusion timestep *t* is projected by an MLP and injected into the block to modulate and integrate information across different diffusion stages. The last convolution is zero-initialized to improve convergence stability and accelerate convergence in large-scale training [21,22]. The attention block (AttenBlock in Figure 5b) adopts a self-attention mechanism, where the correlation weights between the query (*q*) and key (*k*) are computed via softmax and applied to the value (*v*) for adaptive feature aggregation, thereby enhancing the modeling of long-range dependencies and improving spatial representations.

At the deeper, lower-resolution stages of the diffusion backbone, SFHRBAtt, shown in Figure 5c, applies an AttnBlock after SFHRB to incorporate long-range dependencies into the spatial-frequency representation. At the bottleneck between the encoder and decoder, MidBlock, shown in Figure 5d, sequentially stacks a ResBlock, an AttnBlock, and another ResBlock to refine the feature representation and capture global context. Similarly, at the deeper stages of the SAR conditional encoder, AFFRBAtt, shown in Figure 5e, applies self-attention after AFFRB, allowing long-range structural relationships to be modeled on top of the adaptively recalibrated frequency-domain features.

### 3.2. Loss Functions

To effectively train the proposed dual-domain conditional diffusion model, we design a spatial-frequency dual-domain loss that jointly constrains network learning in both the spatial and frequency domains. An MSE loss is used in the spatial domain. In the frequency domain, additional constraints are introduced by applying an $L_{1}$ loss to supervise the spectral representations of both the noise and the target samples. The overall loss is defined as

(16) $$L=L_{s}+\lambda_{1}L_{f}^{\left(1\right)}+\lambda_{2}L_{f}^{\left(2\right)},$$

(17) $$L_{s}={∥\epsilon -Z∥}_{2}^{2},$$

(18) $$L_{f}^{\left(1\right)}={∥\epsilon_{\mathrm{FFT}}-Z_{\mathrm{FFT}}∥}_{1},$$

(19) $$L_{f}^{\left(2\right)}={∥O_{0\_\mathrm{FFT}}-{O^{\prime }}_{0\_\mathrm{FFT}}∥}_{1},$$

where $\lambda_{1}$ and $\lambda_{2}$ are weighting factors that balance the contributions of the two frequency-domain loss terms, and both are set to 0.1 in this work. ϵ denotes the random Gaussian noise added during the forward process, *Z* denotes the noise image predicted by the network, and $\epsilon_{\mathrm{FFT}}$, $Z_{\mathrm{FFT}}$, $O_{0\_\mathrm{FFT}}$ and ${O^{\prime }}_{0\_\mathrm{FFT}}$ are the frequency-domain representations obtained from ϵ, *Z*, $O_{0}$ and $O_{0}^{\prime }$, respectively. $O_{0}^{\prime }$ denotes the target estimate obtained from the predicted noise *Z* via one-step prediction,

(20) $$O_{0}^{\prime }=\frac{O_{t}-\sqrt{1-{\bar{\alpha }}_{t}}\cdot Z}{\sqrt{{\bar{\alpha }}_{t}}}.$$

## 4. Experimental Scheme

### 4.1. Datasets

Two sets of training data were used in this study. One is a public dataset, and the other is newly developed in this study.

- SEN1-2 dataset

The SEN1-2 dataset [23] includes 282,384 image pairs from Sentinel-1 and Sentinel-2 satellites worldwide, each with dimensions of 256 × 256 pixels. These SEN1-2 images also encompass all four seasons of the year, ensuring a diverse and comprehensive sample collection. Sentinel-1 images were acquired in interferometric wide swath (IW) mode as ground-range-detected (GRD) products using VV polarization, while Sentinel-2 provides corresponding optical images in red, green, and blue bands.

- S1S2 dataset

Considering that quantization accuracy provides greater distinction between image details, a new dataset is built for this work, named Sentinel-1 to Sentinel-2 (S1S2). S1S2 has 15 image pairs from different cities using Sentinel-1 and corresponding Sentinel-2 images. The values range from 0 to 10,000. The time difference between SAR and optical data was restricted to no more than one day to mitigate temporal discrepancies. Sentinel-1 images utilized the IW mode of GRD products in dual-polarization (VV and VH) channels, which can achieve better image quality compared to single polarization [24]. The Sentinel-2 images have the red, green, blue, and near-infrared (NIR) bands. Preprocessing of the Sentinel-1 data included orbit correction, thermal noise removal, radiometric calibration, speckle noise removal (with the Refined Lee filter), and terrain correction. The backscattering values of preprocessed SAR images are converted into decibels. The Sentinel-1 images were geographically registered towards Sentinel-2. The data sources for the newly constructed dataset are listed in Table 1. This dataset can be downloaded from https://github.com/isstncu/s1s2 (accessed on 28 June 2026).

Both datasets were divided into training and testing sets. From the spring subset of the SEN1-2 dataset, 6000 image pairs were randomly chosen for the training set, with an additional 500 pairs allocated for testing. Meanwhile, the newly created S1S2 dataset obtained a total of 7150 pairs of 256 × 256-sized image patches without overlapping. Among them, 6000 pairs were randomly selected for training, with 500 pairs designated for testing.

### 4.2. Methods for Comparison

The proposed method, dualCDM, was compared with six state-of-the-art image translation methods, including Attn-CycleGAN (ASGIT) [25], ICGAN [26], IUNet [27], SWIN-GAN (SWIN) [9], CDDPM [2], and SFDiff [14]. All compared methods were implemented using their default parameter settings. The code of the proposed method can be downloaded from https://github.com/isstncu/dualcdm (accessed on 28 June 2026).

The proposed method was trained over 600 epochs with a learning rate of 0.00002. The Adam approach was used as the optimizer with parameter $\beta_{1}=0.9$, $\beta_{2}=0.999$. The input size is 64 × 64. The proposed method randomly cropped each 256 × 256 image into sixteen 64 × 64 patches as the input. The batch size is 48. During inference, the proposed method employed deterministic accelerated sampling with 25 steps, following [28]. Training and testing were carried out on an NVIDIA Tesla A40.

Because GAN-based methods generate an output through a single forward pass, whereas diffusion models rely on iterative denoising, their computational profiles are not directly comparable. We, therefore, limit the complexity analysis to the three diffusion-based methods, namely CDDPM, SFDiff, and dualCDM. Table 2 reports the number of trainable parameters, training configurations, estimated memory consumption at a batch size of 1, and testing time for 500 images. DualCDM has the largest parameter count because of its additional spatial-frequency modeling and adaptive frequency-domain feature recalibration modules. Nevertheless, its estimated per-image memory requirement remains relatively low, mainly because the model processes 64×64 patches, which reduces the memory occupied by intermediate activations.

### 4.3. Metrics

The translated images were evaluated with six complementary metrics covering radiometric error, structural preservation, spectral consistency, and global synthesis quality: RMSE, RASE, SSIM, SAM, Q4 [29], and ERGAS [30]. SSIM was computed on grayscale images converted from the RGB channels. SSIM and Q4 are optimal at 1, whereas RMSE, RASE, SAM, and ERGAS are optimal at 0. Bold in comparison tables indicate the best scores.

## 5. Experimental Results

### 5.1. Qualitative Comparison on Two Datasets

**Figure 6:**
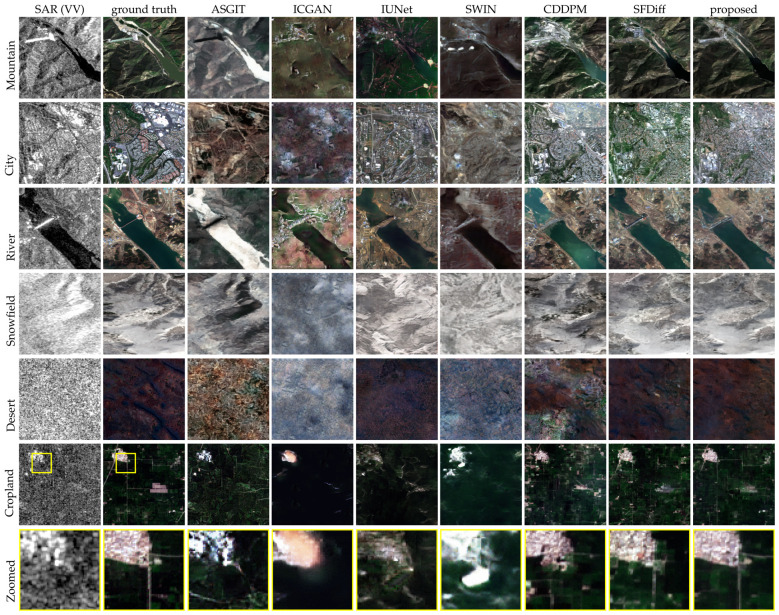
Visual comparison of SAR-to-optical translation results on the SEN1-2 dataset. The yellow boxes indicate the areas to be magnified.

**Figure 7:**
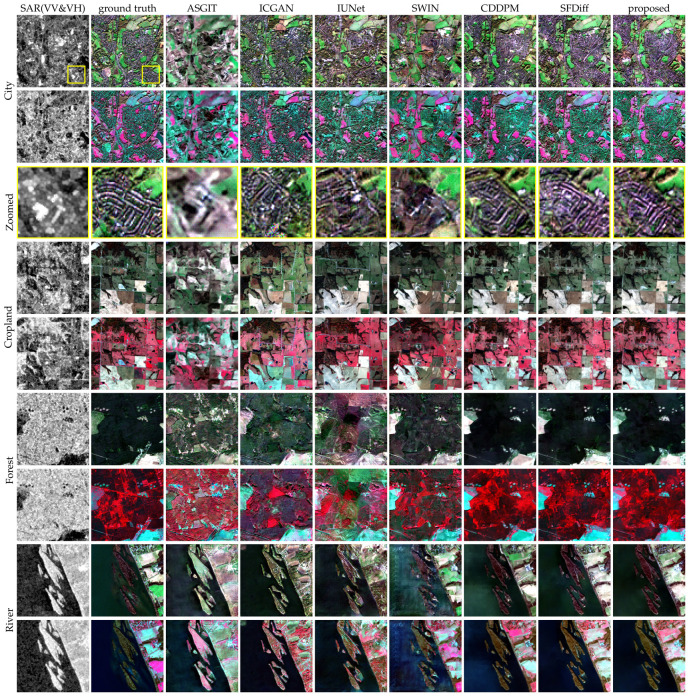
Visual comparison of SAR-to-optical translation results on the S1S2 dataset. The first row represents the red, green, and blue bands of the image. The second row represents the NIR, red, and green bands of the image. The yellow boxes indicate the areas to be magnified.

To better illustrate the performance of our approach, we selected a group of images from the test data and presented the results visually, followed by a qualitative discussion. Figure 6 and Figure 7 show two groups of the selected images from the test data with different characteristics and study areas.

Figure 6 presents representative SAR-to-optical translation results over mountain, city, river, snowfield, desert, and cropland scenes, with all models trained on the SEN1-2 dataset. GAN-based methods produce visually plausible results in some regions, but noticeable artifacts, color shifts, and structural inconsistencies remain in challenging scenes. The diffusion-based methods, particularly CDDPM and SFDiff, generally provide more stable results. SFDiff achieves the strongest visual performance among the competing methods, with improved texture continuity and fewer artifacts than CDDPM in several cases. The proposed method further improves structural coherence and color consistency in most scenes, although some local artifacts remain in particularly ambiguous regions. Owing to space limitations, a detailed enlargement is provided only for the representative cropland scene. The enlarged region shows that the proposed method better preserves the boundaries between fields, roads, and built-up areas while producing a more coherent local color distribution.

Figure 7 presents additional results over cropland, forest, river, and city scenes from the S1S2 dataset. The models use two-channel Sentinel-1 GRD observations (VV and VH) together with multi-band optical references. The additional polarization and spectral information provides richer constraints for SAR-to-optical translation and generally improves the visual quality of the generated images. In urban areas, some GAN-based methods begin to recover clearer geometric patterns than those obtained from single-band inputs, but color distortion and unstable textures are still evident. Diffusion-based approaches produce more consistent color distributions and structural patterns across different land-cover types. Among them, SFDiff and the proposed method achieve comparable overall visual quality, while the proposed method generally provides improved color fidelity and structural coherence. A local enlargement of the representative city scene is included to make these differences more visible. The enlarged comparison shows that the proposed method retains the repetitive road and building patterns more clearly and produces fewer local color inconsistencies than most competing methods.

### 5.2. Quantitative Comparison on the SEN1-2 Dataset

To extend the analysis from qualitative visual inspection to the entire test set, we further conduct a comprehensive quantitative evaluation using multiple metrics computed over all test samples. Table 3 reports the quantitative comparison of different SAR-to-optical translation methods on the SEN1-2 dataset, where the results are averaged over 500 test images. As shown in the table, the proposed method consistently achieves the best performance across all evaluation metrics, including RMSE, SAM, RASE, ERGAS, SSIM, and Q4, indicating superior radiometric accuracy, structural fidelity, and spectral consistency. Among the GAN-based methods, more advanced architectures such as IUNet and SWIN show improved performance. However, their overall performance remains significantly inferior to that of diffusion-based methods, particularly in terms of spectral and structural consistency.

The SEN1-2 dataset contains multiple scene categories, which enables an evaluation of the proposed method under different land cover conditions. We perform scene specific tests on four representative categories, namely cropland, mountain, city, and river. Each category includes 10 test images. The quantitative results are reported in Table 4. The proposed method achieves the best performance in all four categories for most metrics, showing stable advantages in radiometric accuracy, structural fidelity, and spectral consistency. Performance in the city category is generally lower for all methods because the scene contains dense man made structures and complex spatial patterns that are difficult to infer from SAR observations alone. Even in this challenging setting, the proposed method remains the top performer across all metrics.

### 5.3. Quantitative Comparison on the S1S2 Dataset

Similar to the evaluation on the SEN1-2 dataset, we further report the quantitative comparison on the S1S2 dataset in Table 5. Benefiting from the higher radiometric resolution and multi-band optical targets in S1S2, the performance differences among competing methods become more clearly observable. As shown in the table, the proposed method achieves the best results across all metrics, including lower errors (RMSE, SAM, RASE, and ERGAS) and higher perceptual or structural scores (SSIM and Q4), indicating improved radiometric accuracy, spectral consistency, and structural fidelity. Moreover, diffusion-based approaches (CDDPM and SFDiff) consistently outperform GAN-based methods on this dataset, while the proposed method further advances the overall performance beyond the strongest diffusion baseline.

### 5.4. Test on Large Images

To further evaluate the effectiveness of the proposed method and its ability to produce spatially consistent predictions at a larger scale, we conducted an additional large image experiment on the S1S2 dataset. Specifically, we used the 12th image pair, where a 4120 × 5120 region was used for training and a disjoint 1000 × 1000 region was reserved for testing.

**Figure 8:**
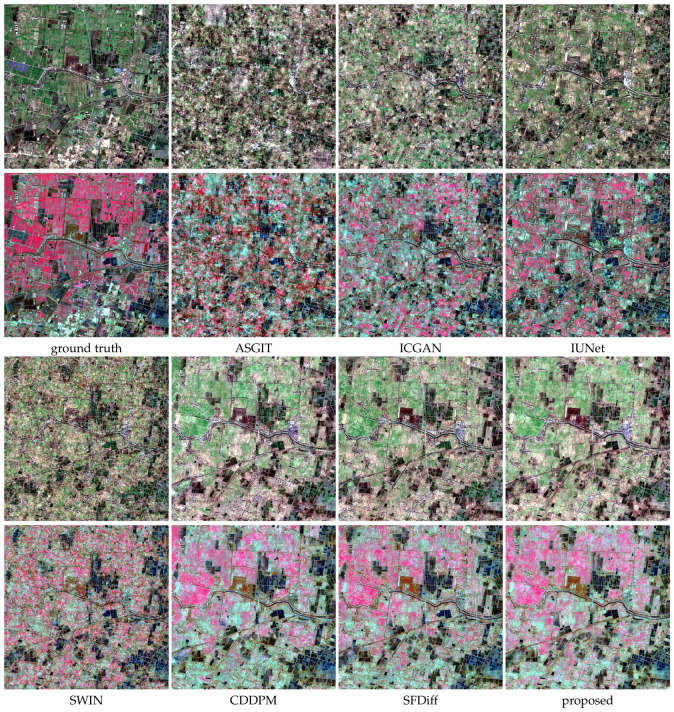
Visual comparison of large image SAR-to-optical translation results on the S1S2 dataset (the first row represents the red, green, and blue bands of the image. The second row represents the NIR, red, and green bands of the image).

The qualitative and quantitative results are reported in Figure 8 and Table 6. As shown, GAN-based methods exhibit noticeable deviations from the ground truth, among which SWIN yields the best performance but still suffers from non-negligible radiometric and structural errors. In contrast, diffusion-based methods provide substantially improved results.

Notably, different from the previous two evaluations, the performance gap between SFDiff and CDDPM becomes smaller in this large image setting, while the proposed method still maintains a clear advantage across all metrics. A possible explanation is that this setting uses only a single large image for training, which substantially reduces sample diversity and limits the coverage of frequency patterns. As a result, SFDiff, whose frequency modeling mainly relies on point-wise modulation at each frequency, may have limited capacity to learn a reliable spectral prior and to generalize under such restricted training data. In contrast, the proposed method explicitly models cross-frequency and cross-channel correlations in the frequency domain, making it less sensitive to reduced data diversity and enabling more stable improvements in radiometric fidelity and structural consistency.

### 5.5. Statistical Significance Analysis

Although dualCDM generally improves the visual quality, it is not uniformly superior to the competing methods in every local region. For example, the mountain scene in Figure 6 still contains a bright granular artifact in the upper-left region, similar to that observed in the CDDPM result. In the river scenes in Figure 7, some areas retain SAR-derived spatial patterns and exhibit insufficient spectral transformation over water and shoreline regions. These cases indicate that challenging scattering conditions may still lead to local artifacts or incomplete modality conversion. Therefore, the qualitative comparisons alone are insufficient to establish a consistent advantage across the complete test set.

To determine whether the overall improvements are statistically reliable rather than being driven by a limited number of favorable samples, we performed a paired comparison between dualCDM and SFDiff, the strongest baseline in the quantitative evaluation. The six metrics were calculated separately for each test image, yielding paired observations for the two methods. A two-sided Wilcoxon signed-rank test was adopted because normality could not be assumed for the per-image metric differences. The resulting *p*-values were adjusted across the six metrics using the Holm procedure. We additionally report 95% bootstrap confidence intervals for the paired mean differences and rank-biserial correlations as effect sizes. A difference was considered statistically significant when the Holm-adjusted *p*-value was below 0.05.

The results are summarized in Table 7. On both SEN1-2 and S1S2, dualCDM significantly outperforms SFDiff across all six metrics. All adjusted *p*-values are below 0.05, and none of the 95% confidence intervals includes zero. The rank-biserial correlations range from 0.6031 to 0.9771 on SEN1-2 and from 0.6229 to 0.9975 on S1S2, indicating that the improvements are both statistically significant and broadly consistent across the test images. The strongest consistency is observed for SSIM, for which dualCDM outperforms SFDiff on 97.2% of the SEN1-2 samples and 99.6% of the S1S2 samples. These results show that, despite several local limitations, the overall performance gains are not determined by a small number of favorable cases.

### 5.6. Ablation Study

To validate the rationality and effectiveness of the proposed SFHRB and AFFRB modules, as well as the role of the two frequency-domain loss functions during training, we conduct a systematic and comprehensive ablation study on the S1S2 dataset. To expedite the ablation comparisons, all variants are trained for only 100 epochs and evaluated on the entire test set of S1S2. The results are summarized in Table 8.

First, by comparing Column 1 with Columns 2 and 3, we observe that incorporating frequency-domain learning substantially improves the overall quality of SAR-to-optical translation. The results further indicate that the time modulation mechanism is crucial for effective frequency-domain feature modeling in diffusion models. Second, by comparing Column 1 with Columns 4 and 5, we find that performing frequency-domain denoising on the conditional SAR input helps improve the translation results. Meanwhile, representing the phase using cos(Φ) and sin(Φ) effectively mitigates representation ambiguity and numerical instability caused by phase discontinuities. The results in Column 6 further demonstrate that jointly modeling the above two designs yields more pronounced improvements across multiple metrics. In addition, comparing Column 1 with Columns 7 and 8 shows that both the frequency-domain loss on noise and the frequency-domain loss on the one-step reconstruction can consistently enhance translation performance, where the latter brings a larger gain. Column 9 suggests that jointly optimizing the two frequency-domain losses leads to further improvements across all metrics. Finally, Column 10, which integrates the SFHRB module, the AFFRB module, and both frequency-domain losses, achieves the best performance on all evaluation metrics, thereby validating the synergistic effectiveness of the proposed modules and loss designs.

### 5.7. Visualization and Interpretation of SFHRB and AFFRB

**Figure 9:**
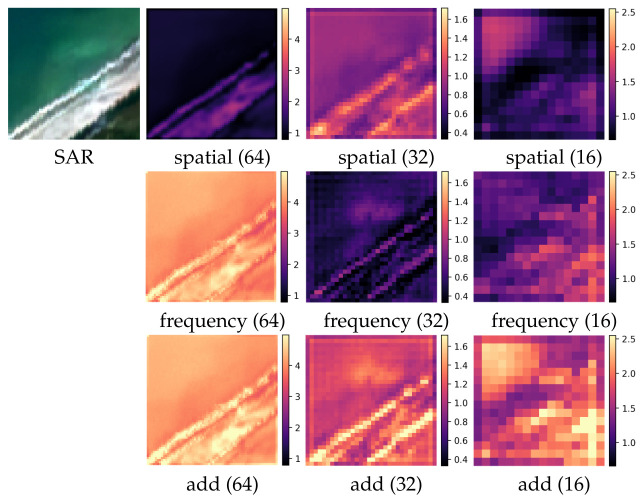
Multi-scale spatial-frequency feature decomposition and fusion in SFHRB.

Figure 9 visualizes the spatial branch, frequency branch, and their fused representations in SFHRB at three feature resolutions. At the 64×64 level, the spatial branch mainly responds to localized boundaries and fine-scale structures, whereas the frequency branch produces broader responses around the dominant diagonal structure. Their fusion retains local details while introducing more spatially continuous responses. At the 32×32 level, the frequency branch highlights several coherent and approximately parallel structures that are less evident in the spatial branch, and the fused representation integrates these responses with the locally extracted spatial features. At the 16×16 level, both branches mainly represent coarse regional patterns, while their combination provides a more complete description of the dominant large-scale structure. These observations indicate that the spatial and frequency branches learn complementary rather than redundant representations. The 8×8 level is omitted because its spatial resolution is too coarse for meaningful structural interpretation.

**Figure 10:**
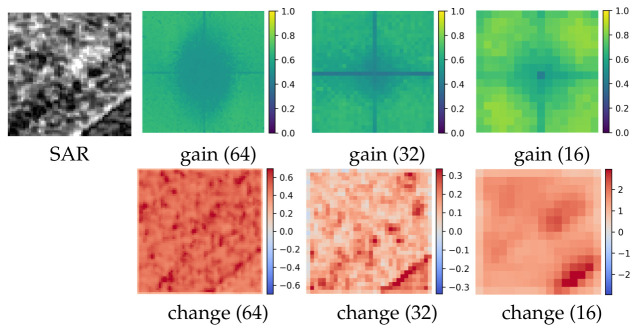
Visualization of learned spectral gains and multi-scale feature-response changes induced by AFFRB.

Figure 10 illustrates the frequency-dependent modulation learned by AFFRB and its effect on multi-scale SAR feature representations. The spectral gain maps are clearly non-uniform and vary across encoder levels, showing that AFFRB does not apply a fixed attenuation factor or a predefined frequency filter. At the 64×64 level, the response-change map shows a broad enhancement of fine-scale local variations. At the 32×32 level, the enhanced responses become more spatially selective and are concentrated around several linear and boundary-like patterns. At the 16×16 level, the response changes mainly correspond to broader regional structures, including the prominent diagonal feature in the lower-right region. These results indicate that the frequency-attention-guided AFFRB performs scale-dependent spectral recalibration and translates it into spatially varying refinement of SAR features.

## 6. Discussions: Generalization Performance

**Figure 11:**
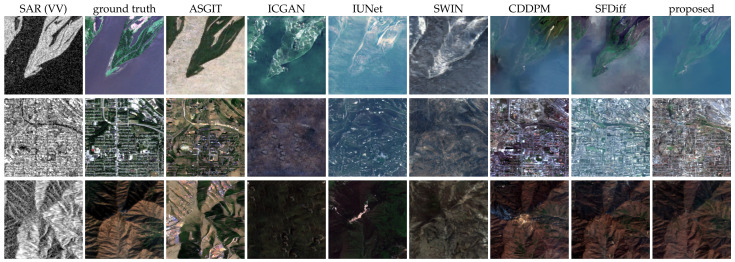
Visual comparison of cross-region and cross-season generalization on the SEN1-2 dataset.

To further evaluate the generalization capability of the proposed method, we conduct additional experiments on test data that differ from the training conditions. Specifically, 150 test images are selected from the SEN1-2 dataset, covering three seasons other than spring, which is exclusively used for training. In this way, the evaluation reflects both seasonal variations and regional diversity that were not observed during training. The quantitative and qualitative results are summarized in Table 9 and Figure 11, respectively.

As expected, due to differences in geographic regions and acquisition times, the performance of all compared methods degrades to some extent. Such variations introduce changes in illumination conditions, vegetation states, and surface properties, which pose additional challenges for SAR-to-optical translation. Nevertheless, the proposed method consistently achieves the best performance across most evaluation metrics and produces visual results that are closest to the ground truth optical images. Both the quantitative scores and visual comparisons demonstrate that the proposed method exhibits stronger robustness to seasonal and regional changes, indicating superior generalization ability compared with competing methods.

## 7. Conclusions

In this study, we proposed dualCDM, a conditional diffusion framework for SAR-to-optical image translation that jointly models spatial- and frequency-domain information. In the diffusion backbone, SFHRB combines a spatial convolution branch with complex-valued convolution in the Fourier domain. By aggregating neighboring Fourier coefficients across input feature channels, the frequency branch captures local cross-frequency and cross-channel interactions, while timestep modulation adapts the learned responses to different diffusion stages. In the SAR conditional encoder, AFFRB predicts input-dependent real-valued gains from the magnitude and trigonometric phase representations of intermediate GRD features. This design provides adaptive frequency-domain recalibration without introducing an additional phase shift, while the residual connection preserves the original conditional information. The spatial-frequency dual-domain objective further constrains both noise prediction and one-step optical reconstruction, improving structural and frequency-domain consistency.

We also constructed the S1S2 dataset using 16-bit Sentinel-2 reflectance data, retaining the original 0–10,000 value range and including the near-infrared band. Experiments on SEN1-2 and S1S2 demonstrate that dualCDM improves radiometric accuracy, spectral consistency, and structural preservation over representative GAN- and diffusion-based methods. Paired statistical analyses further show significant improvements over SFDiff, the strongest competing method, across all six evaluation metrics on both datasets. Nevertheless, the qualitative results indicate that dualCDM is not uniformly superior in every local region, and artifacts or incomplete modality conversion can still occur in scenes with complex terrain, strong backscatter, or ambiguous water and shoreline responses. Future work will, therefore, focus on improving the robustness of the conditional representation under these challenging scattering conditions.

## Figures and Tables

**Table 1:** Data sources of the new dataset (all captured in 2022).

Date		City	Lat	Lon	Image Size
S1	S2				
07.19	07.19	Gniezeno (Poland)	52.5	17.3	6400 × 6400
09.07	09.07	Alexandria (Romania)	43.1	25.4	6400 × 6400
09.21	09.22	Paris (France)	48.6	2.6	6400 × 6400
10.19	10.18	Dnipro (Ukraine)	48.5	35.0	6400 × 6400
10.28	10.28	Chicago (United States)	40.3	−94.8	6400 × 6400
10.31	11.01	Walcourt (Belgium)	50.3	4.3	6400 × 6400
11.03	11.03	Warsaw (Poland)	52.6	21.5	3840 × 3840
11.04	11.04	Patras (Greece)	39.3	22.1	2560 × 2560
11.12	11.13	Nijmegen (Netherlands)	51.8	5.6	3840 × 3840
11.25	11.25	Birmingham (United Kingdom)	52.7	−2.2	5120 × 5120
11.29	11.28	New South Wales (Australia)	−29.4	149.8	5120 × 5120
12.18	12.18	Suqian (China)	30.4	113.4	5120 × 5120
12.18	12.18	Suceava (Romania)	47.7	26.1	6400 × 6400
12.22	12.21	Lancaster (United States)	40.1	−76.3	6400 × 6400
12.30	12.31	Shumen (Bulgaria)	43.4	27.3	5120 × 5120

**Table 2:** Model complexity and computational efficiency among diffusion-based methods.

	CDDPM	SFDiff	DualCDM
Params (M)	60.14	116.72	135.14
Est. memory (GB)	4.75	7.30	2.97
Time/500 (s)	8500	9998	10,023
Time/image (s)	17.000	19.996	20.046

**Table 3:** Quantitative evaluation of SAR-to-optical translation on the SEN1-2 dataset.

Model	RMSE	SAM	RASE	ERGAS	SSIM	Q4
ASGIT	69.2071	0.1760	1.0178	1.0997	0.1623	0.2377
ICGAN	55.7127	0.1744	0.7105	0.7359	0.2088	0.1985
IUNet	53.2892	0.1686	0.7119	0.7465	0.2097	0.2018
SWIN	53.1838	0.1676	0.6469	0.6635	0.2288	0.1808
CDDPM	50.7611	0.1271	0.6399	0.6568	0.2402	0.2409
SFDiff	40.0003	0.1180	0.4872	0.4962	0.2828	0.3582
proposed	**37.7443**	**0.1116**	**0.4600**	**0.4686**	**0.3273**	**0.4014**

**Table 4:** Quantitative evaluation across different land cover scenes on the SEN1-2 dataset.

Model	RMSE	SAM	RASE	ERGAS	SSIM	Q4
Corpland						
ASGIT	70.6232	0.3424	1.8512	1.9737	0.1371	0.2681
ICGAN	54.3168	0.3104	1.3274	1.4036	0.2279	0.2316
IUNet	47.0659	0.2858	1.1622	1.2150	0.2547	0.2015
SWIN	45.4925	0.3067	1.1034	1.1578	0.3087	0.2292
CDDPM	47.8810	0.2365	1.2615	1.3319	0.2917	0.3021
SFDiff	34.4848	**0.2075**	0.8571	0.8936	0.3527	0.4610
proposed	**32.5384**	0.2114	**0.8025**	**0.8392**	**0.4000**	**0.4892**
Mountain						
ASGIT	52.0851	0.1617	0.6581	0.6672	0.2184	0.3311
ICGAN	48.8418	0.1191	0.5896	0.5903	0.1754	0.2440
IUNet	54.5209	0.1336	0.6874	0.6873	0.1961	0.3190
SWIN	45.8874	0.1217	0.5656	0.5682	0.1941	0.2489
CDDPM	41.3723	0.0866	0.5043	0.5066	0.3024	0.4555
SFDiff	32.7870	0.0801	0.3990	0.3994	0.3376	0.5870
proposed	**30.9919**	**0.0796**	**0.3785**	**0.3788**	**0.3971**	**0.6231**
City						
ASGIT	76.0168	0.1325	1.1341	1.1423	0.0678	0.2420
ICGAN	56.0254	0.1435	0.8512	0.8623	0.1177	0.1564
IUNet	57.3062	0.1235	0.8585	0.8678	0.0945	0.1233
SWIN	50.0540	0.1377	0.7251	0.7337	0.1354	0.1688
CDDPM	54.1109	0.1028	0.7648	0.7692	0.1236	0.1639
SFDiff	46.2064	0.1140	0.6459	0.6488	0.1564	0.2726
proposed	**43.4053**	**0.0991**	**0.6071**	**0.6101**	**0.1837**	**0.2907**
River						
ASGIT	88.5326	0.2355	1.4709	1.5404	0.2330	0.3901
ICGAN	61.9619	0.2261	1.0143	1.0299	0.3115	0.3365
IUNet	56.5494	0.2227	0.9162	0.9268	0.3799	0.3016
SWIN	55.0803	0.2212	0.9299	0.9523	0.3896	0.3440
CDDPM	39.7251	0.1229	0.6797	0.6970	0.4964	0.5388
SFDiff	31.8373	**0.1038**	0.5292	0.5432	0.5705	0.6573
proposed	**30.5516**	0.1091	**0.5050**	**0.5170**	**0.5909**	**0.6819**

**Table 5:** Quantitative evaluation of SAR-to-optical translation on the S1S2 dataset.

Model	RMSE	SAM	RASE	ERGAS	SSIM	Q4
ASGIT	784.0077	0.1840	0.3805	0.3085	0.4768	0.2681
ICGAN	523.4226	0.0988	0.2543	0.2085	0.5259	0.3994
IUNet	519.8615	0.0987	0.2527	0.2085	0.5258	0.4085
SWIN	534.7400	0.1056	0.2575	0.2162	0.5092	0.3920
CDDPM	477.4949	0.0854	0.2306	0.1953	0.5985	0.4467
SFDiff	418.2357	0.0754	0.2025	0.1666	0.6235	0.5141
proposed	**401.4297**	**0.0728**	**0.1943**	**0.1591**	**0.6507**	**0.5369**

**Table 6:** Quantitative evaluation of large image testing on the S1S2 dataset.

Model	RMSE	SAM	RASE	ERGAS	SSIM	Q4
ASGIT	526.7946	0.1154	0.2494	0.2026	0.7659	0.2054
ICGAN	486.6729	0.1056	0.2304	0.1915	0.7480	0.2227
IUNet	515.6891	0.1087	0.2442	0.2055	0.7046	0.2264
SWIN	453.8826	0.0992	0.2149	0.1801	0.7433	0.2398
CDDPM	409.0849	0.0833	0.1937	0.1612	0.8141	0.3156
SFDiff	405.8978	0.0834	0.1922	0.1603	0.8075	0.3311
proposed	**385.9193**	**0.0811**	**0.1827**	**0.1531**	**0.8205**	**0.3471**

**Table 7:** Statistical significance analysis of dualCDM against SFDiff on the SEN1-2 and S1S2 datasets.

Dataset	Metric	SFDiff	DualCDM	Δ	95% CI of Δ	Adjusted *p*	$r_{\mathrm{rb}}$	Win Rate (%)
SEN1-2	RMSE	40.0003	**37.7443**	2.2560	[1.8984, 2.6084]	$3.66\times 10^{-44}$	0.7235	82.2
	SAM	0.1180	**0.1116**	0.0063	[0.0048, 0.0078]	$1.51\times 10^{-31}$	0.6031	76.6
	RASE	0.4872	**0.4600**	0.0272	[0.0213, 0.0323]	$1.05\times 10^{-44}$	0.7299	82.2
	ERGAS	0.4962	**0.4686**	0.0276	[0.0214, 0.0333]	$1.05\times 10^{-44}$	0.7291	82.2
	SSIM	0.2828	**0.3273**	0.0450	[0.0420, 0.0481]	$3.78\times 10^{-79}$	0.9771	97.2
	Q4	0.3582	**0.4014**	0.0432	[0.0368, 0.0496]	$1.28\times 10^{-34}$	0.6363	75.0
S1S2	RMSE	418.2357	**401.4297**	16.806	[14.9782, 18.6364]	$1.17\times 10^{-57}$	0.8296	86.6
	SAM	0.0754	**0.0728**	0.0026	[0.0022, 0.0029]	$8.25\times 10^{-45}$	0.7275	79.6
	RASE	0.2025	**0.1943**	0.0082	[0.0073, 0.0090]	$6.23\times 10^{-58}$	0.8326	86.6
	ERGAS	0.1666	**0.1591**	0.0075	[0.0068, 0.0083]	$6.69\times 10^{-62}$	0.8619	88.6
	SSIM	0.6235	**0.6507**	0.0270	[0.0259, 0.0281]	$1.91\times 10^{-82}$	0.9975	99.6
	Q4	0.5141	**0.5369**	0.0228	[0.0192, 0.0264]	$1.55\times 10^{-33}$	0.6229	74.8

Note: The paired difference Δ is defined such that a positive value consistently indicates better performance of dualCDM. The *p*-values are obtained using a two-sided Wilcoxon signed-rank test and adjusted across the six metrics within each dataset using the Holm procedure. $r_{\mathrm{rb}}$ denotes the rank-biserial correlation, and the win rate is the percentage of test images for which dualCDM outperforms SFDiff.

**Table 8:** Ablation experiment.

SFHRB (without time modulation)		✓								
SFHRB			✓			✓				✓
AFFRB (FreqAttn-$\mathrm{concat}\left[M,\Phi \right]$)				✓						
AFFRB (FreqAttn-$\mathrm{concat}\left[M,\cos(\Phi ),\sin(\Phi )\right]$)					✓	✓				✓
$L_{f}^{\left(1\right)}$							✓		✓	✓
$L_{f}^{\left(2\right)}$								✓	✓	✓
RMSE	497.0738	493.1099	486.5744	493.7522	491.5883	482.3787	481.0528	460.4320	458.7144	449.2712
SAM	0.0861	0.0859	0.0850	0.0855	0.0854	0.0837	0.0836	0.0829	0.0820	0.0802
RASE	0.2409	0.2389	0.2357	0.2393	0.2382	0.2336	0.2331	0.2230	0.2221	0.2175
ERGAS	0.2039	0.2013	0.1977	0.2023	0.2011	0.1963	0.1942	0.1847	0.1837	0.1795
SSIM	0.5912	0.5908	0.5983	0.5912	0.5932	0.6007	0.6078	0.6010	0.6107	0.6150
Q4	0.4318	0.4355	0.4450	0.4361	0.4366	0.4491	0.4666	0.4737	0.4797	0.4946

**Table 9:** Quantitative evaluation of cross-region and cross-season generalization on the SEN1-2 dataset.

Model	RMSE	SAM	RASE	ERGAS	SSIM	Q4
ASGIT	65.7333	0.2043	0.8901	0.9540	0.1420	**0.1890**
ICGAN	60.3954	0.2093	0.7983	0.8634	0.1970	0.1363
IUNet	60.9791	0.2109	0.7972	0.8352	0.1987	0.1332
SWIN	57.5210	0.1989	0.7622	0.7983	0.2269	0.1336
CDDPM	61.6716	0.1923	0.8704	0.9172	0.1875	0.1315
SFDiff	56.7792	**0.1896**	0.7689	0.8056	0.2137	0.1340
proposed	**53.4720**	**0.1896**	**0.6884**	**0.7185**	**0.2531**	0.1542

## Data Availability

The original contributions presented in this study are included in the article. Further inquiries can be directed to the corresponding author.
